# Alteration of Antiviral Signalling by Single Nucleotide Polymorphisms (SNPs) of Mitochondrial Antiviral Signalling Protein (MAVS)

**DOI:** 10.1371/journal.pone.0151173

**Published:** 2016-03-08

**Authors:** Fei Xing, Tomoh Matsumiya, Ryo Hayakari, Hidemi Yoshida, Shogo Kawaguchi, Ippei Takahashi, Shigeyuki Nakaji, Tadaatsu Imaizumi

**Affiliations:** 1 Department of Vascular Biology, Institute of Brain Science, Hirosaki University Graduate School of Medicine, Hirosaki, Japan; 2 Department of Gastroenterology and Hematology, Hirosaki University Graduate School of Medicine, Hirosaki, Japan; 3 Department of Social Medicine, Hirosaki University Graduate School of Medicine, Hirosaki, Japan; Duke University Medical Center, UNITED STATES

## Abstract

Genetic variation is associated with diseases. As a type of genetic variation occurring with certain regularity and frequency, the single nucleotide polymorphism (SNP) is attracting more and more attention because of its great value for research and real-life application. Mitochondrial antiviral signalling protein (MAVS) acts as a common adaptor molecule for retinoic acid-inducible gene-I (RIG-I)-like receptors (RLRs), which can recognize foreign RNA, including viral RNA, leading to the induction of type I interferons (IFNs). Therefore, MAVS is thought to be a crucial molecule in antiviral innate immunity. We speculated that genetic variation of MAVS may result in susceptibility to infectious diseases. To assess the risk of viral infection based on MAVS variation, we tested the effects of twelve non-synonymous MAVS coding-region SNPs from the National Center for Biotechnology Information (NCBI) database that result in amino acid substitutions. We found that five of these SNPs exhibited functional alterations. Additionally, four resulted in an inhibitory immune response, and one had the opposite effect. In total, 1,032 human genomic samples obtained from a mass examination were genotyped at these five SNPs. However, no homozygous or heterozygous variation was detected. We hypothesized that these five SNPs are not present in the Japanese population and that such MAVS variations may result in serious immune diseases.

## Introduction

Innate immune responses are the first line of defence that protects the host from viral invasion. These responses are triggered by recognition of the pathogen-associated molecular patterns (PAMPs) of pathogens via pattern recognition receptors (PRRs) [1]. Retinoic acid-inducible gene-I (RIG-I)-like receptors (RLRs), including RIG-I [2], melanoma differentiation-associated gene-5 (MDA-5) [3] and laboratory of genetics and physiology 2 (LGP2) [4], are such PRRs and are expressed in various types of cells. RLRs can recognize foreign double-stranded RNA (dsRNA) in the cytoplasm and transmit the signal to downstream molecules, leading to the induction of type I interferons (IFNs) [5]. Following viral recognition, RLRs bind to their unique adaptor molecule, namely, mitochondrial antiviral signalling protein (MAVS) [6], which is also known as virus-induced signalling adaptor (VISA) [7], IFN-β promoter stimulator-1 (IPS-1) [8], and caspase activation and recruitment domain adaptor inducing IFN-β (Cardif) [9]. MAVS receives signals from RIG-I or MDA-5 and then activates IFN regulatory factor 3 (IRF3) and nuclear factor-κB (NF-κB) through TANK-binding kinase 1 (TBK1) and IκB kinases (IKKs), respectively [10]. Activated IRF3 and NF-κB subsequently translocate into the nucleus and coordinately initiate the transcription of type I IFNs [11].

MAVS consists of 540 amino acids and has three main domains, namely an N-terminal caspase recruitment and activation domain (CARD), an internal proline-rich region and a C-terminal transmembrane (TM) domain [11], which allows MAVS to localize to the outer mitochondrial membrane. MAVS receives antiviral signals by binding to RIG-I or MDA-5 through its CARD domain [12]. Subsequently, MAVS transduces the signal to downstream molecules through its proline-rich region. The distribution of MAVS in the mitochondria is vital for its role in antiviral signalling, as dissociation from the mitochondria due to deletion of the TM domain results in ablation of the signalling [13].

Studies have reported that several viruses escape from PRR-dependent antiviral responses by degrading MAVS [14–16]. Kawai et al. also confirmed that loss of MAVS blocked IFN induction by viral infection [8]. In a previous study, we also found that overexpression of MAVS enhanced IFN-β induction in response to dsRNA [17]. All of these findings reveal that there is a positive correlation between the amount of MAVS and its antiviral role. However, little is known about the effect of the quality of MAVS on infectious diseases.

Variations of immune-related molecules are associated with increased severity of infectious diseases. For example, patients with alterations in glutamic acid 223 of NF-κB essential modulator (NEMO) show recurrent sinopulmonary infections or necrotizing soft-tissue methicillin-resistant *Staphylococcus aureus* (MRSA) infections and *Streptococcus anginosus* subdural empyema with bacteraemia [18]. Therefore, we speculate that variations in the MAVS gene may result in susceptibility to infectious diseases.

In this study, we aimed to analyse the association of MAVS single nucleotide polymorphisms (SNPs) with infectious diseases to assess the risk of viral infection based on MAVS variation. We screened twelve MAVS SNPs from the National Center for Biotechnology Information (NCBI) database, five of which were selected for genotyping because of their obvious effects on antiviral signalling in cell experiments. We also genotyped 1,032 Japanese genomic samples at these five SNPs. Unexpectedly, the five SNPs were not found in the Japanese population that we tested. In any case, we suggest that such MAVS variations may result in serious immune diseases, especially in infections.

## Materials and Methods

### Cell culture and treatment

HeLa cells (JCRB Cell Bank, Osaka, Japan) and stably MAVS-silenced 293-flp cells (293-flp MAVS KD cells, generated in our previous study [19]) were maintained in a 5% CO_2_ atmosphere at 37°C in Dulbecco’s modified Eagle’s medium (DMEM) (Sigma-Aldrich, St. Louis, MO) supplemented with 10% fetal bovine serum (FBS) (Perbio Science, Switzerland) and antibiotics (Invitrogen, Carlsbad, CA).

### Plasmid construction and site-directed mutagenesis

To obtain the MAVS-wild type (WT) cloning vectors, the SalI-NotI site of the cDNA in pCMV-Myc-MAVS (generated in our previous study [17]) was transferred into the cloning vector pBlueScript II SK(+) (Fermentas, Canada). MAVS SNP-cDNAs were generated by site-directed mutagenesis using the MAVS-WT-encoding vector pBlueScript II SK(+) as the template as well as the primer pairs shown in Table 1.

**Table 1:** Primer sets for the syntheses of MAVS-SNP-cDNAs.

SNP ID		primer
rs78448735	sense	ATACCCTTCAGCGGTGGCCCGGCTGGGTG
	antisense	CACCCAGCCGGGCCACCGCTGAAGGGTAT
rs76715450	sense	CTGACCTCCAGCAGGCATCAGGAGC
	antisense	GCTCCTGATGCCTGCTGGAGGTCAG
rs62640879	sense	GCTCTGAGAATAGGAGCCTTGGGTCGGAG
	antisense	CTCCGACCCAAGGCTCCTATTCTCAGAGC
rs45563035	sense	GCCTGTCCAGGAGAGCCAGGCGCCAGAGT
	antisense	ACTCTGGCGCCTGGCTCTCCTGGACAGGC
rs45529136	sense	CCAACACCCGCCAGCGCCACTGGAG
	antisense	CTCCAGTGGCGCTGGCGGGTGTTGG
rs45437096	sense	GCCTCACACCATCCTGTGGGCCTGTGTCT
	antisense	AGACACAGGCCCACAGGATGGTGTGAGGC
rs34591263	sense	CGGCACTGAGGGGCATTGAGCTAGTTGATC
	antisense	GATCAACTAGCTCAATGCCCCTCAGTGCCG
rs17857295	sense	TGGCCTCTGTCTACGAGAGCTACCAGCCT
	antisense	AGGCTGGTAGCTCTCGTAGACAGAGGCCA
rs11908032	sense	CGGCACTGAGGGGCAGTGAGCTAGTTGAT
	antisense	ATCAACTAGCTCACTGCCCCTCAGTGCCG
rs11905552	sense	GGCACTGAGGGGCTTTGAGCTAGTTGATC
	antisense	GATCAACTAGCTCAAAGCCCCTCAGTGCC
rs7269320	sense	GCTAGACAGCAGCTTTGAGAATAGGGGCC
	antisense	GGCCCCTATTCTCAAAGCTGCTGTCTAGC
rs7262903	sense	GCGGGCATCAGGAGAAGGACACAGAACTG
	antisense	CAGTTCTGTGTCCTTCTCCTGATGCCCGC

To obtain the MAVS SNP expression vectors, the SalI-NotI site of the cDNAs in the pBlueScript II SK(+)-MAVS-SNPs were transferred into the mammalian expression vector pCMV-myc (Clontech, Mountain View, CA).

To obtain positive controls for SNP genotyping by quantitative PCR, fragments of the WT genomic sequence that were less than 200 bp in length and that contained each SNP site were amplified from genomic DNA in A549 cells using GoTaq Green Master Mix (Promega) and the primer pairs listed in Table 2.

**Table 2:** Primer sets for obtaining positive controls for SNP genotyping.

SNP ID		primer
rs78448735	sense	AACCGGGACACCCTCTG
	antisense	CCCTCAGTGCCGCAATG
rs76715450	sense	GAGTCCTCCTCTGACCTG
	antisense	CACCTTGTCTCCTGTTCA
rs45437096	sense	CAAGGTAATGGTCTTTGG
	antisense	GCCAGTAGATACAACTGA
rs11908032	sense	GGGTGGAGTACTTCATTG
	antisense	GAGGATGACAGAGAAAGG
rs11905552	sense	GGGTGGAGTACTTCATTG
	antisense	GAGGATGACAGAGAAAGG
rs671	sense	TGGTGGCTACAAGATGTCGG
	antisense	CCCCAACAGACCCCAATCC

The amplified products were then inserted into the cloning vector pTAC-1 using a TA PCR Cloning Kit (BioDynamics Laboratory Inc.) following the manufacturer’s protocol. SNP-including genomic sequences were generated by site-directed mutagenesis using each SNP site-containing or WT genomic fragment-encoding vector as the template as well as the primer pairs shown in Table 3.

**Table 3:** Primer sets for the syntheses of SNP-including genomic sequences.

SNP ID		primer
rs78448735	sense	CTTCAGCGGTGGCCCGGCT
	antisense	AGCCGGGCCACCGCTGAAG
rs76715450	sense	ACCTCCAGCAGGCATCAGG
	antisense	CCTGATGCCTGCTGGAGGT
rs45437096	sense	ACACCATCCTGTGGGCCTG
	antisense	CAGGCCCACAGGATGGTGT
rs11908032	sense	CTGAGGGGCAGTGAGCTAG
	antisense	CTAGCTCACTGCCCCTCAG
rs11905552	sense	CTGAGGGGCTTTGAGCTAGTT
	antisense	AACTAGCTCAAAGCCCCTCAG
rs671	sense	GCATACACTAAAGTGAAAA
	antisense	TTTTCACTTTAGTGTATGC

cDNAs encoding full-length tumour necrosis factor (TNF) receptor-associated factor (TRAF) 2 and TRAF6 were amplified from cDNAs isolated from HeLa cells using Phusion DNA Polymerase (Finnzymes, Keilaranta, Finland) and the primers NotI-TRAF2-F (5’-CTTgcggccgcGATGGCTGCAGCTAGCGTGAC-3’) and SalI-TRAF2-R (5’-TCTAGAgtcgacTTAGAGCCCTGTCAGGTCCA-3’) or NotI-TRAF6-F (5’-CTTgcggccgcGATGAGTCTGCTAAACTGTGA-3’) and SalI-TRAF6-R (5’-TCTAGAgtcgacCTATACCCCTGCATCAGTAC-3’), respectively. Each amplified product was inserted into the SalI and NotI sites of the mammalian expression vector p3xFLAG (Sigma-Aldrich).

The plasmids were purified using a plasmid purification kit (Qiagen, Hilden, Germany). All DNA constructs were analysed by DNA sequencing.

### Transfection

Transient transfection of HeLa or 293-flp MAVS KD cells was performed as previously reported [20]. Briefly, these cells were seeded at a density of 1.5×10^5^ or 3×10^5^ cells per well, respectively, in 12-well culture plates for 16 to 20 h before transfection to reach 70 to 80% confluence.

To overexpress exogenous MAVS, TRAF2, and TRAF6, the cells were transfected with the MAVS expression vectors or an empty control vector using Lipofectamine LTX (Invitrogen). To introduce the foreign dsRNA polyinosinic-polycytidylic acid (poly I:C) (Sigma-Aldrich), the cells were transfected using Tranfectin (Bio-Rad). These cells were then incubated for the indicated period of time, depending on the experiment.

### Quantitative RT-PCR

Total RNA was extracted from the cells using an illustra RNAspin Mini RNA Isolation Kit (GE Healthcare, Piscataway, NJ). The total RNA (500 ng) served as a template for single-strand cDNA synthesis in a reaction using an oligo(dT)_18_ primer and M-MLV reverse transcriptase (Invitrogen) under the conditions indicated by the manufacturer. A CFX96 real-time PCR detection system (Bio-Rad, Hercules, CA) was used for the quantitative analyses of IFN-β, MAVS and 18S rRNA. The sequences of the primers were as follows:

IFN-β-F (5’-CCTGTGGCAATTGAATGGGAGGC-3’),

IFN-β-R (5’-CCAGGCACAGTGACTGTACTCCTT-3’),

MAVS-F (5’-ATAAGTCCDGAGGGCACCTTT-3’),

MAVS-R (5’-GTGACTACCAGCACCCCTGT-3’),

18S rRNA-F (5’-ACTCAACACGGGAAACCTCA-3’), and

18S rRNA-R (5’-AACCAGACAAATCGCTCCAC-3’).

The amplification reactions were performed with SsoFast EvaGreen Supermix (Bio-Rad) according to the manufacturer’s specifications. The amplification conditions were as follows: 30 s at 98°C, followed by heating consecutively at 98°C and 58°C for 5 s each for 40 cycles. After the amplification was complete, a melting curve was generated by slowly heating from 65°C to 95°C at 0.5°C increments, with 5 s per step, with continuous monitoring of the fluorescence. Quantitative analyses of the data were performed using CFX Manager (Bio-Rad).

### Enzyme-linked immunosorbent assay (ELISA)

Conditioned culture medium was collected at the indicated times and was centrifuged at 12,000 x*g* for 5 min at 4°C to remove cell debris. The IFN-β concentration in the culture medium was then measured using a human IFN-β ELISA kit (Kamakura Techno-Science, Japan).

### Immunoblot analyses

After two washes with phosphate-buffered saline (PBS; pH 7.4), cells were lysed in hypotonic lysis buffer (10 mM Tris (pH 7.4), 100 mM NaCl, 1.5 mM MgCl_2_, and 0.5% NP-40) containing 0.2% protease inhibitors. The cell lysate was cleared by centrifugation at 12,000 x*g* for 5 min at 4°C, after which 10 μg of the lysate was subjected to electrophoresis on a 7.5% SDS-polyacrylamide gel. To observe phosphorylated MAVS, the lysate was separated on a 10% SuperSep^™^ Phos-tag^™^ polyacrylamide gel (Wako, Japan).

The proteins were then transferred to polyvinylidene fluoride (PVDF) membranes (Millipore, Billerica, MA), which were subsequently blocked for 1 h at room temperature in TBST buffer (20 mM Tris (pH 7.4), 150 mM NaCl, and 0.1% Tween 20) containing 5% nonfat dry milk (blocking buffer). Next, the membranes were incubated overnight at 4°C with one of the following primary antibodies: mouse anti-Cardif (MAVS) (Enzo Life Sciences, Miami, FL) or mouse anti-Myc (Clontech). After five washes with TBST, the membranes were further incubated for 1 h at room temperature with a ZyMax anti-mouse IgG antibody (Invitrogen) coupled with horseradish peroxidase (HRP) at a 1:10,000 dilution in blocking buffer. For detection of immunoprecipitated Myc- and FLAG-tagged proteins, anti-Myc-HRP (MBL Life Science, Japan) and anti-FLAG-HRP (Sigma-Aldrich) antibodies, respectively, were used.

The washes were repeated using TBST, and then the immunoreactive bands were visualized using the Luminata Crescendo Western HRP Substrate (Millipore).

### Immunoprecipitation (IP)

Cells were washed twice with ice-cold PBS and lysed in IP lysis buffer (20 mM Tris (pH 7.4), 100 mM NaCl, 1.5 mM MgCl_2_, and 1% Triton-X 100) containing protease inhibitor cocktail (Sigma-Aldrich). Thirty minutes after incubation on ice, the lysates were centrifuged at 6,500 x*g* for 15 min at 4°C, and the supernatants were collected in fresh tubes. To immunoprecipitate Myc-tagged MAVS protein, the supernatants were incubated with Myc beads (MBL Life Science, Japan) for 1 h at 4°C. After extensive washing with ice-cold Myc-IP wash buffer (50 mM Tris-HCl (7.5), 150 mM NaCl, and 0.05% NP-40), the immunoprecipitates were eluted by boiling the beads in 2 X SDS-PAGE sample buffer.

### Immunofluorescence analyses

HeLa cells that were grown on glass coverslips were incubated with MitoTracker Orange CMTMRos (Molecular Probes) for 30 min and then fixed with 4% formaldehyde for 20 min, permeabilized with 0.1% Triton X-100 for 10 min and blocked with 3% BSA for 1 h. The cells were subsequently incubated for 1 h with mouse monoclonal anti-Myc. After a washing step, the cells were incubated with Alexa 488-conjugated anti-mouse IgG (Invitrogen). Afterwards, the cells were mounted with ProLong Gold Antifade Reagent (Invitrogen), and the subcellular localizations of MAVS and the mitochondria were visualized by confocal laser scanning microscopy (C1si, Nikon, Japan).

### Genomic samples

A total of 1,032 human genomic samples were obtained from the Iwaki Health Promotion Program, which is mainly organized by the Department of Social Medicine, Hirosaki University (Japan). All of these genomic samples were extracted from the leucocytes of native inhabitants of the Iwaki region of Hirosaki city (Japan) who participated in health examinations as part of that programme. This study was approved by the ethics committee of the Hirosaki University Graduate School of Medicine. The subjects were informed about the goals of the study and provided written informed consent before the collection of DNA samples.

### SNP genotyping

Genomic samples were genotyped at the rs78448735, rs76715450, rs45437096, rs11908032 and rs11905552 SNPs in the MAVS gene and at rs671 in the aldehyde dehydrogenase 2 (ALDH2) gene. PCR-based genotyping was performed using a CFX384 real-time PCR detection system (Bio-Rad, Hercules, CA) and TaqMan assays or high-resolution melting (HRM).

Primer pairs and fluorescently labelled probe pairs were designed for each SNP in the TaqMan assays (Table 4).

**Table 4:** Primers sets and probes for TaqMan assays.

SNP ID		primer	probe
rs78448735	sense	AACCGGGACACCCTCTG	CTTCAGCGGTGGTCCGGCTGG
	antisense	CCCTCAGTGCCGCAATG	CTTCAGCGGCGGCCCGGCT
rs76715450	sense	GAGTCCTCCTCTGACCTG	TGACCTCCAGCGGGCATCAGGA
	antisense	CACCTTGTCTCCTGTTCA	TGACCTCCAGCAGGCATCAGGA
rs45437096	sense	CAAGGTAATGGTCTTTGG	ACACCATCCCGTGGGCCT
	antisense	GCCAGTAGATACAACTGA	CACACCGTCCTGTGGGCCTGT
rs11908032	sense	GGGTGGAGTACTTCATTG	ACTGAGGGGCTGTGAGCTAG
	antisense	GAGGATGACAGAGAAAGG	ACTGAGGGGCAGTGAGCTAG
rs11905552	sense	GGGTGGAGTACTTCATTG	TGAGGGGCTGTGAGCTAGTT
	antisense	GAGGATGACAGAGAAAGG	CTGAGGGGCTCTGAGCTAGTT

PCR reactions (10 μL) consisting of genomic DNA (100 ng), primers (10 μM), probes (4 μM), SsoAdvanced Universal Probes Supermix (Bio-Rad) and distilled H_2_O were performed in white Hard-Shell 384-well plates (Bio-Rad). The PCR conditions were as follows: heating for 3 min at 95°C, followed by heating consecutively at 95°C for 15 s and 67.9°C (rs78448735), 67.2°C (rs76715450) or 63.1°C (rs45437096 and rs11908032) for 1 min for 44 cycles. The data were analysed using CFX Manager (Bio-Rad).

The primer pairs used for HRM are shown in Table 5.

**Table 5:** Primers sets for HRM.

SNP ID		primers
rs78448735	sense	AACCGGGACACCCTCTG
	antisense	CCCTCAGTGCCGCAATG
rs76715450	sense	TCTGACCTGGCAGCCCTC
	antisense	AGTTCTGTGTCCTGCTCCTG
rs45437096	sense	GACCTCCAGCCTCACACC
	antisense	CAGGGGCTGGAAGGAGAC
rs11908032	sense	GGAGTACTTCATTGCGGCAC
	antisense	GTAGACAGAGGCCACTTCGT
rs11905552	sense	GGAGTACTTCATTGCGGCAC
	antisense	GTAGACAGAGGCCACTTCGT
rs671	sense	TGGTGGCTACAAGATGTCGG
	antisense	CCCCAACAGACCCCAATCC

PCR reactions (10 μL) consisting of genomic DNA (100 ng), primers (10 μM), Precision Melt Supermix (Bio-Rad) and distilled H_2_O were run in white Hard-Shell 384-well plates (Bio-Rad). The PCR conditions were as follows: heating for 2 min at 95°C, followed by heating consecutively at 95°C for 10 sec and 62.3°C for 1 min for 40 cycles. After heating for 30 s at 95°C and 1 min at 40°C, an HRM curve was generated by gradual heating from 75°C to 90°C at 0.1°C (rs78448735, rs76715450, rs45437096, and rs671) or 0.2°C (rs11905552) increments for 10 s per step. Then, another HRM curve was generated by gradual heating from 75°C to 90°C at 0.2°C increments for 10 s (rs78448735, rs76715450, rs45437096, and rs671) or 1 s (rs11905552) per step after heating for 30 s at 95°C and 1 min at 40°C. Continuous monitoring of fluorescence was performed along with melting curve generation, and an analysis of the data was performed using Precision Melt Analysis Software (Bio-Rad).

### Statistics

Statistical analyses were performed using Student’s t-test. Differences were considered significant at P<0.05.

## Results

### Genetic variation in human MAVS

Variations in the human MAVS gene were investigated using the NCBI SNP database and we chose twelve MAVS SNPs for our study, eight of which were reported in a study from another group while we were performing the current study [21].

**Fig 1:**
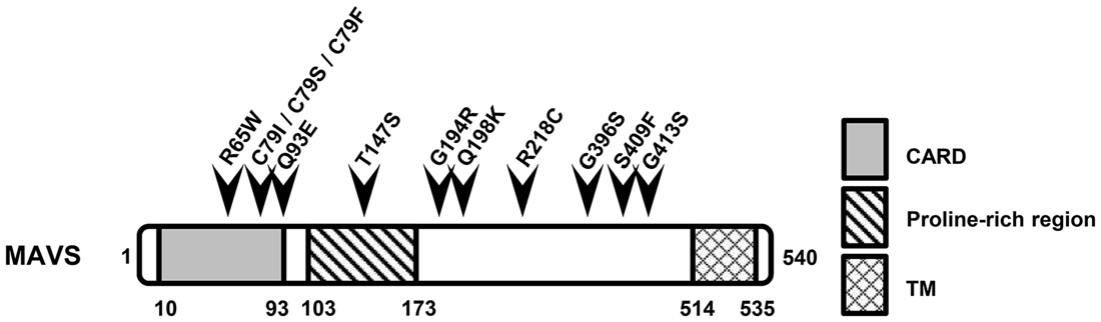
SNPs selected in this study. Schematic diagram of the conserved domains of MAVS and the locations of SNPs.

All of these SNPs were in the coding region and caused amino acid substitutions; these SNPs are most likely to be associated with human diseases. We excluded SNPs in promoter region because the transcriptional regulatory site of the MAVS gene is incompletely understood. Multiple variants as well as in-del variants were also excluded to focus on better understanding the effect of the single variants on the function of MAVS. The information of SNPs studied here is shown in Fig 1 and Table 6. The NCBI database revealed that these SNPs are not conserved in the mouse genome.

**Table 6:** SNPs analysed in this study.

gene	SNP ID	position	allele change	residue change
MAVS	rs78448735	CDS	CGG⇒TGG	R65W
MAVS	rs76715450	CDS	GGG⇒AGG	G194R
MAVS	rs62640879	CDS	GGC⇒AGC	G413S
MAVS	rs45563035	CDS	ACC⇒AGC	T147S
MAVS	rs45529136	CDS	GGC⇒AGC	G396S
MAVS	rs45437096	CDS	CGT⇒TGT	R218C
MAVS	rs34591263	CDS	TGT⇒ATT	C79I
MAVS	rs17857295	CDS	CAG⇒GAG	Q93E
MAVS	rs11908032	CDS	TGT⇒AGT	C79S
MAVS	rs11905552	CDS	TGT⇒TTT	C79F
MAVS	rs7269320	CDS	TCT⇒TTT	S409F
MAVS	rs7262903	CDS	CAG⇒AAG	Q198K
ALDH2	rs671	CDS	GAA⇒AAA	E504K

### Effect of MAVS SNPs on protein modification of MAVS

**Fig 2:**
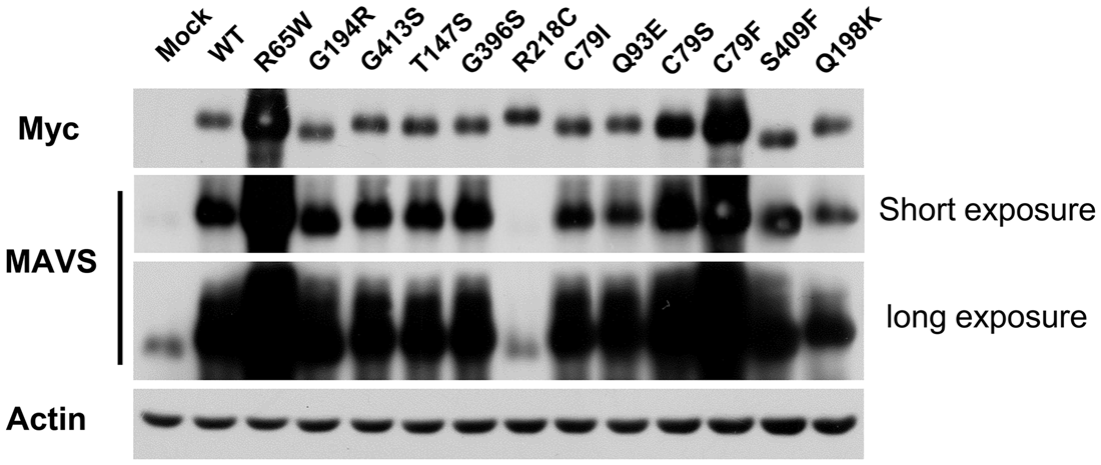
Influence of SNPs on MAVS protein. HeLa cells were transfected with an empty plasmid (mock) or a plasmid encoding WT or variant MAVS and were then incubated for 24 h. Cell extracts were subsequently subjected to SDS-PAGE and blotted with anti-MAVS or anti-Myc antibody. The results are representative of three independent experiments.

Because quality MAVS protein (e.g. a truncated form of MAVS) is essential for MAVS-mediated antiviral signalling [22], we initially investigated the influence of MAVS SNPs on the levels of protein and modification of MAVS. Following transient transfection of HeLa cells with WT or MAVS SNP encoding vectors, the expression of exogenous MAVS proteins were confirmed by immunoblotting (IB) (Fig 2). R65W and C79F increased the levels of MAVS protein, suggesting that these SNPs may contribute to the stability of MAVS protein. It appeared that G194R and S409F migrated faster than WT MAVS. In contrast, R218C was associated with slow migration of MAVS protein. R218C was not detected with the anti-MAVS antibody that we used, suggesting that the antigenicity of MAVS was altered. Note that all of the MAVS SNP vectors were sequenced and confirmed to have no nucleotide insertion or deletion and to have no mutation(s) except the MAVS SNPs in their coding regions. These results indicated that MAVS SNPs may affect posttranscriptional and/or posttranslational modification (PTM) of MAVS. Protein phosphorylation is the most conventional PTM, so we investigated whether MAVS SNPs alter the level of phosphorylation of MAVS. The result from Phos-tag PAGE showed slow migration of the WT MAVS and SNPs that we tested (S1 Fig), demonstrating that these MAVS proteins were constitutively phosphorylated under unstimulating conditions. The patterns of migration were quite similar to those in SDS-PAGE, indicating that the phosphorylation was unlikely to alter the molecular weights of the MAVS SNPs.

### MAVS SNPs alter intracellular distribution of MAVS

**Fig 3:**
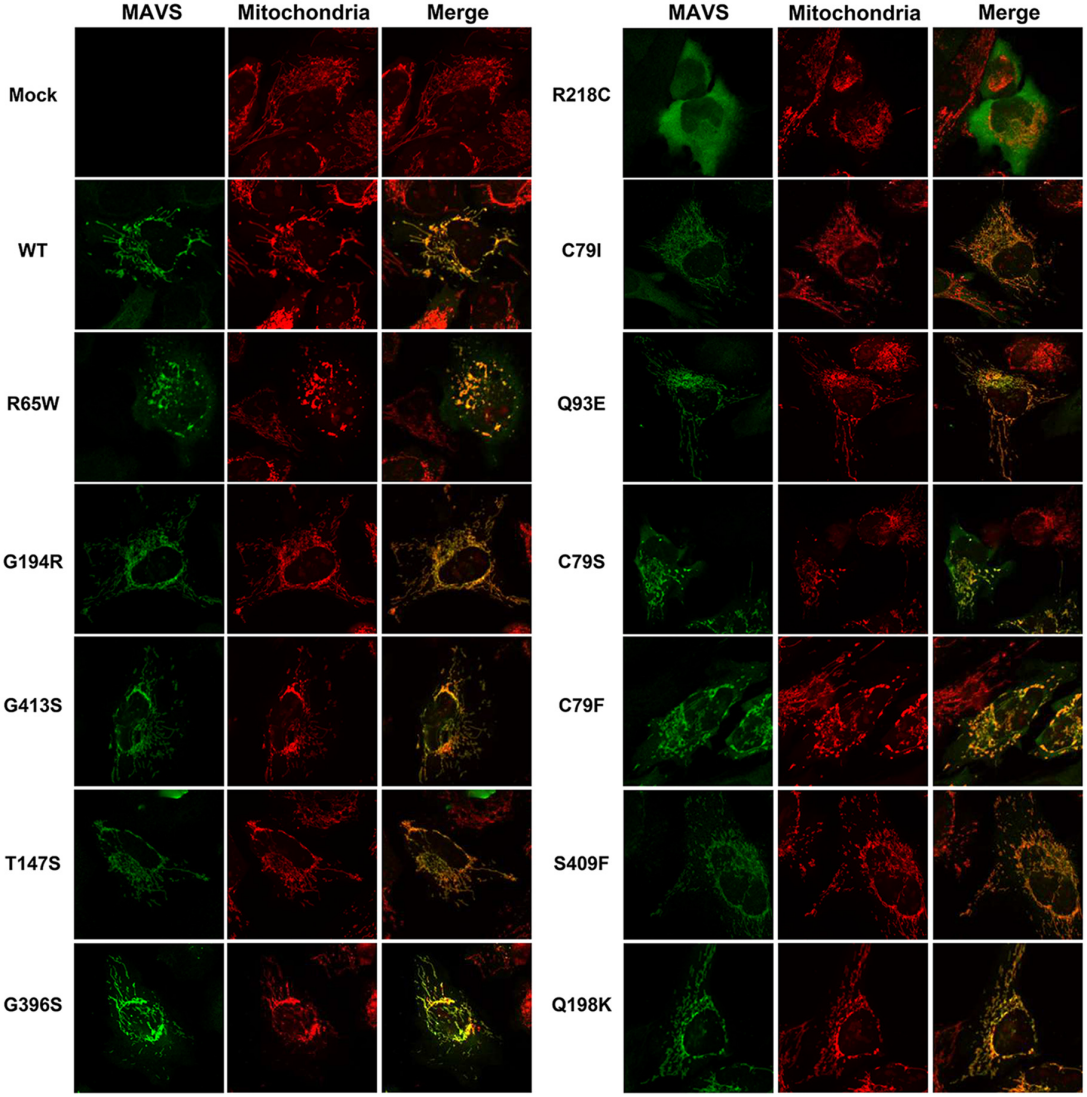
Effects of SNPs on the intracellular MAVS distribution and mitochondrial morphology. HeLa cells were transfected with an empty plasmid (mock) or a plasmid encoding WT or variant MAVS and were then incubated for 24 h. After treatment with MitoTracker Orange for 30 min, the cells were fixed with 4% paraformaldehyde and incubated with anti-MAVS antibody. MAVS protein was then detected using a secondary antibody coupled to Alexa 488 (MAVS, green).

Intracellular distribution in the mitochondria is vital for the role of MAVS in antiviral signalling [23]. As a result, we next examined the effects of MAVS SNPs on intracellular localization. As reported previously, exogenous WT MAVS as well as endogenous MAVS was located on mitochondria (Fig 3). G194R, G413S, T147S, G396S, C79I, Q93E, S409F, and Q198K did not alter the morphology of mitochondria or the mitochondrial localization of MAVS (Fig 3). In contrast, R218C completely altered the distribution of MAVS from on mitochondria to inside the cytoplasm (Fig 3). R65W, C79S, and C79F induced either mitochondrial fragmentation or mitochondrial aggregation; however, these SNPs did not alter the mitochondrial localization of MAVS (Fig 3). These results suggested that several MAVS SNPs can affect the function of MAVS.

### Altered antiviral signalling by MAVS SNPs

**Fig 4:**
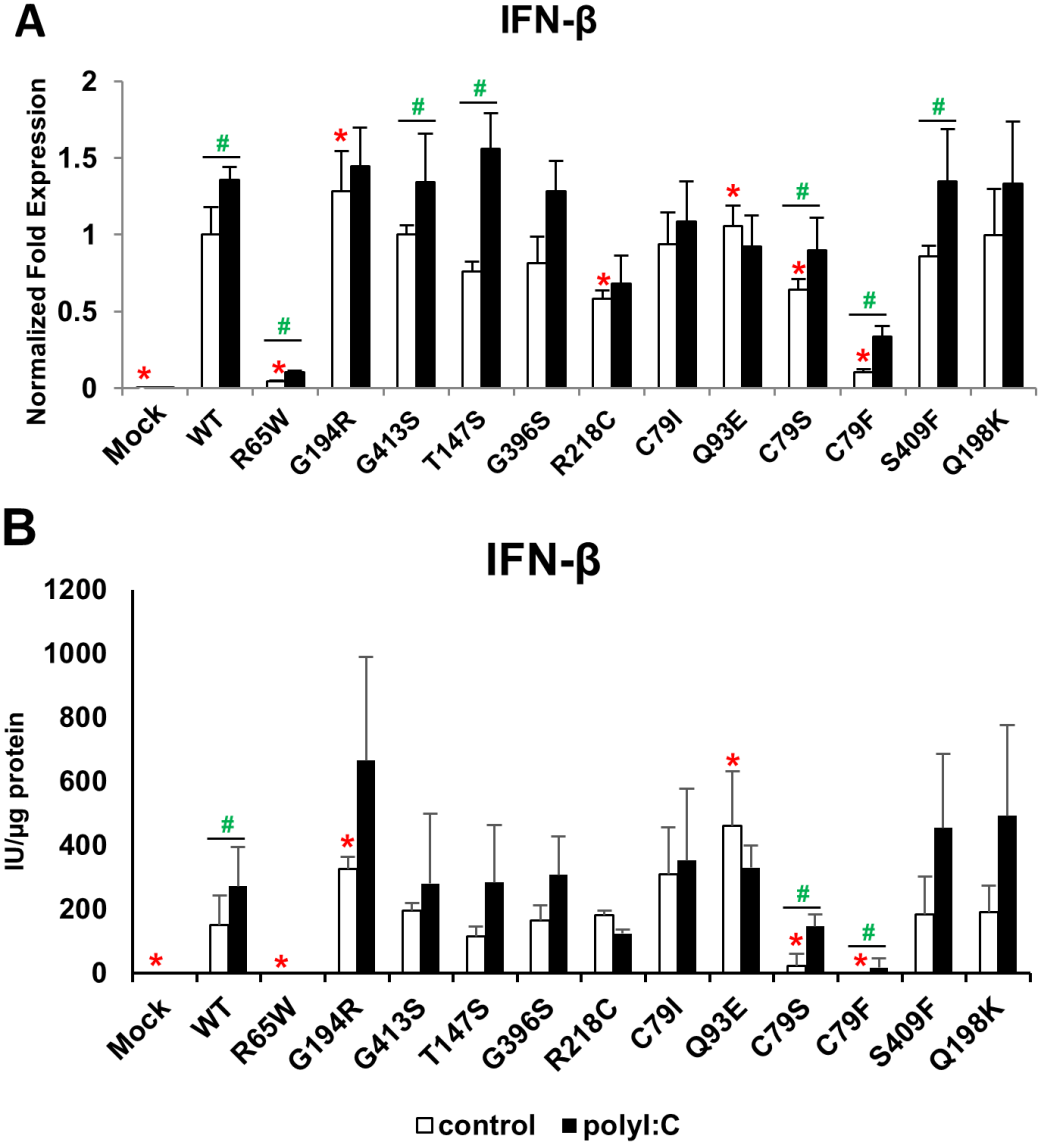
Effects of MAVS SNPs on antiviral signalling in response to poly I:C. Twenty-four hours after transfection with a mock, WT or variant MAVS expression plasmid, stably MAVS-silenced 293-flp cells were further transfected with poly I:C (500 ng/well) for 8 h. A. The mRNA levels of IFN-β were determined using quantitative RT-PCR. B. The levels of IFN-β protein in the culture medium were analysed by ELISA. The data are presented as the mean ± SD of three independent experiments. *P<0.05 vs WT MAVS; #P<0.05 vs poly I:C transfection.

Because MAVS is a crucial molecule in RNA-mediated antiviral signalling, we next examined the effects of variant MAVS proteins on antiviral signalling in response to poly I:C, a synthetic dsRNA analogue. To avoid the impact of endogenous MAVS, we used 293-flp MAVS KO cells [19] and then, exogenous variant MAVS proteins were overexpressed in these cells. In this cell system, poly I:C was not able to induce either mRNA or protein expression of IFN-β due to silencing of MAVS (Fig 4A and 4B). Overexpression of WT MAVS enhanced the induction of IFN-β, and rescued the expression of IFN-β in response to poly I:C. Overexpression of G194R and Q79E significantly enhanced basal- IFN-β expression. G194R did not influence the alteration of IFN-β expression in response to poly I:C. In contrast, Q93E failed to upregulate IFN-β in response to poly I:C.

The effect of G413S, T147S, G396S, C79I, S409F, and Q198K on induction of IFN-β showed a similar pattern compared with that of WT MAVS and did not lead to marked alteration of the IFN-β expression in response to poly I:C. Overexpression of R218C lowered IFN-β expression more than WT MAVS did, and R218C as well as Q93E failed to upregulate IFN-β in response to poly I:C. C79I was able to induce IFN-β mRNA to the same extent as WT MAVS was, but inhibited the protein production of IFN-β. Finally, overexpression of R65W as well as C79F did not induce IFN-β and was able to induce low levels of IFN-β in response to poly I:C.

**Fig 5:**
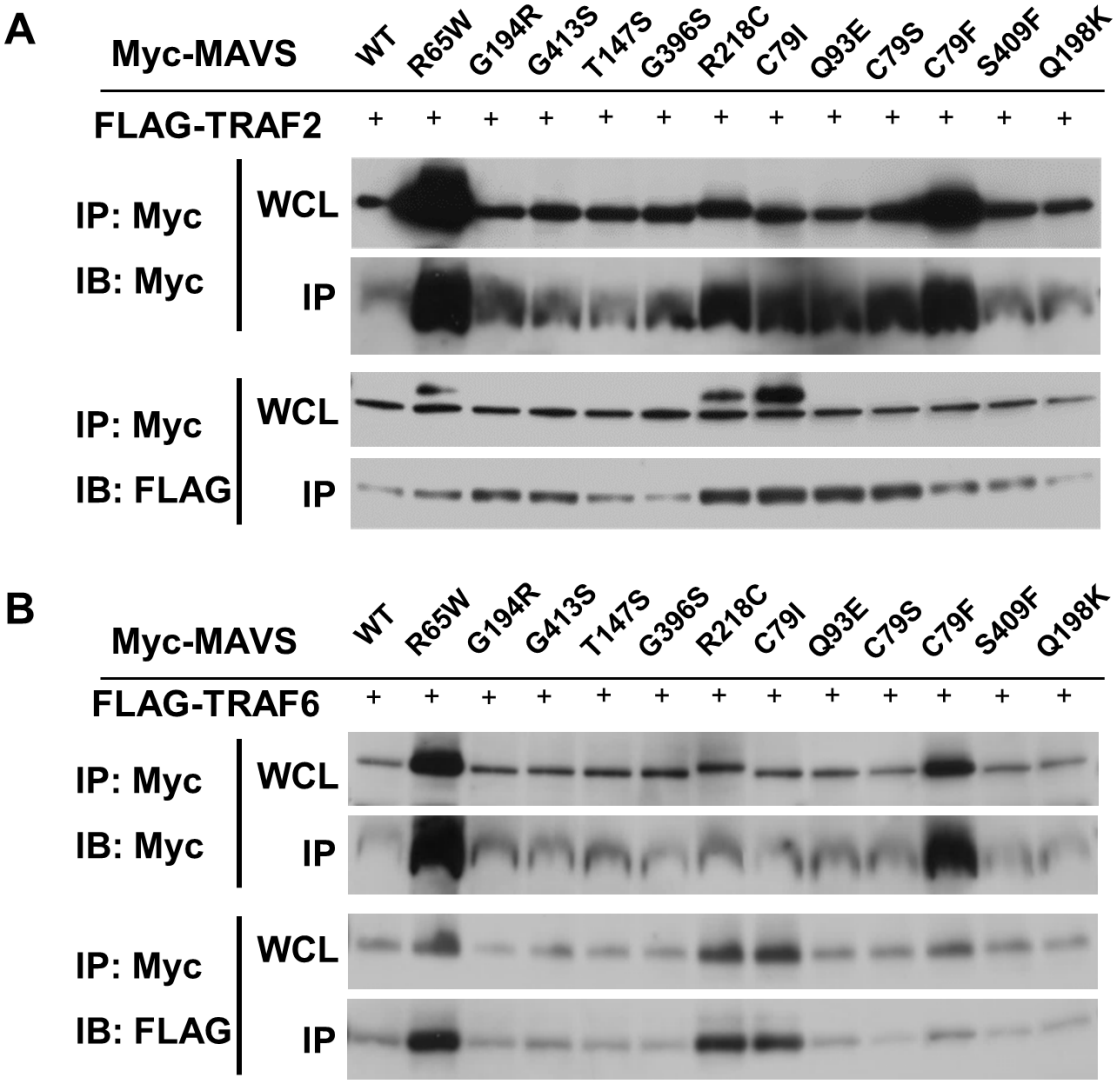
Effects of MAVS SNPs on association with RIG-I, TRAF2, and TRAF6. Twenty-four hours after co-transfection with a Myc-tagged WT or variant MAVS expression plasmid and p3xFLAG-TRAF2 (A) or p3xFLAG-TRAF6 (B), cell lysates were immunoprecipitated with Myc beads. The eluted proteins were then analysed by IB with an antibody against FLAG or Myc, as indicated. The WCL represents 3% of the starting materials used for IP.

To further explore the effect of MAVS SNPs on RLR signalling, we initially examined the association of RIG-I-CARD, an active form of RIG-I [2], with MAVS SNPs and observed no marked alteration of the interaction compared with what was observed for WT MAVS (data not shown). We next examined whether MAVS SNPs affect the interaction of TRAF2 and TRAF6, which have been shown to be associated with MAVS in both the presence and the absence of non-self RNA [6,7]. As reported, a co-IP assay showed that WT MAVS was able to weakly interact with both TRAF2 and TRAF6 (Fig 5A and 5B). We also measured the amount of TRAF2 in whole-cell lysates (WCLs) and compared it with the amount of co-precipitated TRAF2. It appeared that R218C, C79I, Q93E, and C79F enhanced the interaction of MAVS with TRAF2 (Fig 5A). Although the levels of TRAF6 in WCLs from R65W, R218, and C79I were higher than the levels in other lysates, comparative analysis revealed that R65W exhibited relatively more interaction with TRAF6 (Fig 5B).

### Analysis of MAVS SNPs

We performed genotyping of the R65W, G194R, R218C, C79S and C79F SNPs, because these variants were considered to alter the functions of MAVS, as shown by our experimental data above. A total of 1,032 Japanese genomic samples were analysed for these five variants. Unexpectedly, we observed neither homozygous variation nor heterozygous variation for these MAVS SNPs (Table 7).

**Table 7:** Genotype frequencies detected in this study.

gene	SNP ID	genotype frequency (%)		
MAVS	rs78448735	CC	CT	TT
		100 (1032)	0	0
	rs76715450	GG	GA	AA
		100 (1032)	0	0
	rs45437096	CC	CT	TT
		100 (1032)	0	0
	rs11908032	TT	TA	AA
		100 (1032)	0	0
	rs11905552	GG	GT	TT
		100 (1032)	0	0
ALDH2	rs671	GG	GA	AA
		65 (222)	33 (113)	2 (8)

To exclude the potential impact of the quality of these samples, we performed genotyping of rs671 in the ALDH2 gene, which affects dinking behaviour in the Japanese population, in these samples [24]. The frequency of rs671 in the ALDH2 gene detected in this study was 18.5% (Table 7), similar to published data in the NCBI database (http://www.ncbi.nlm.nih.gov/projects/SNP/snp_ref.cgi?rs=671). This result showed that there was no issue regarding the quality of the genomic samples. Collectively, these results suggested that the Japanese population may not express these five MAVS SNPs.

## Discussion

Variants of innate immune system-associated molecules are reported to associate with various clinical diseases including cancer [25], infectious diseases [26] and autoimmune diseases [27]. Hundreds of articles have focused on the genetic variation in the members of Toll-like receptor (TLR) family which recognize various components of pathogens on the cell surface or in intracellular vesicles. However reports about RLR- signalling polymorphisms are much less common. So far, polymorphisms in RIG-I have been shown to be associated with resistance to type I diabetes [28], a decreased antibody level in response to rubella vaccination [29] and modification of the innate immune response of human dendritic cells [30]. Polymorphisms in Ibo, a molecule downstream of MAVS, were also identified to be involved in spontaneous hepatitis C virus (HCV) infection resolution in two independent cohorts [31]. Therefore, we initially thought that variants in the MAVS gene should result in dysfunction of the innate immune system, especially in the infectious diseases due to the gene’s crucial role in antiviral signalling. However, only four articles about MAVS polymorphisms have been published until now. Two of these reports showed an association between MAVS SNPs and systemic lupus erythematosus (SLE) in African-American and Chinese populations [21,32]. Moreover, Marzari et al. found that two SNPs in the MAVS gene showed a modest association with the onset age of multiple sclerosis (MS) patients [33]. Additionally, His et al. have recently reported an interesting association between MAVS SNPs and the symptomatology of Rift Valley Fever (RVF) [34]. According to that report, a SNP in the 3’untranslated region of MAVS is significantly associated with eye symptoms in RVF. The authors also showed a weak correlation between SNPs in the coding region of MAVS and positive serology for RVF. Although they showed no mechanisms of how the SNPs in MAVS influence the clinical symptoms of RVF, their reuslts supported our hypothesis in the present study: that is, that the SNPs in MAVS are functionally associated with susceptibility to viral infection.

Here, we first reported that the R65W and R218C SNPs exerted inhibitory effects on antiviral signalling in response to dsRNA. The R65W variant is located in the CARD domain of MAVS; therefore this variant may affect the interaction of MAVS and RIG-I through alterations in surface structure and hydrophobicity [31]. Mutation in the CARD domain also affects the significantly reduced interaction of MAVS with TRAF3, as shown by co-IP assays [21]. SNPs that are located in the CARD domain, such as the variants R65W, C79S, and C79F, may alter mitochondrial function because of their associated morphological changes (Fig 3). Mitochondrial dynamics modulate RLR signalling; in particular, mitochondrial fragmentation by silencing of Mfn1 or OPA1 significantly inhibits RLR signalling [35]. Within mitochondrial dynamics, MAVS is thought to be a regulator of Mfn1, given that silencing of MAVS induces mitochondrial elongation and that overexpression of MAVS promotes mitochondrial fission [35]. Therefore, our data may show that these SNPs in MAVS dysregulate Mfn1, resulting in the suppression of RLR signalling. Another SNP, R218C, which also inhibits antiviral signalling, is located in an unknown domain in MAVS protein. Although R218C is not within the TM domain, this variant possibly contributes to dislocation of MAVS from the outer mitochondria membrane. Consistent with this, an early reconstitution study clearly showed that dislocation of MAVS from the mitochondria leads to loss of RLR signalling [6]. PTM is the chemical modification of a protein after its translation to form the mature product [35], and PTM is believed to be an essential process in cell signalling. Because PTM affects the molecular weight of a target protein, such a modified protein migrates faster or slower in gel electrophoresis. Over 200 protein PTMs have been described; among those, phosphorylation is arguably the most pervasive, as it is involved in nearly every cellular process [36]. Several reports also indicate the important role of MAVS phosphorylation in RLR signalling. The Polo-box domain (PBD) of the mitotic Polo-like kinase PLK1 negatively regulates antiviral signalling induced by the association with MAVS. This association is reported to require constitutive phosphorylation of the threonine 234 residue of MAVS [37]. In addition, a very recent report has shown that phosphorylation of the serine 442 residue of MAVS is essential for RLR signalling [38]. Protein analysis of phosphorylated protein has shown that G194R, S409F, and R218C do not alter the levels of constitutively phosphorylated MAVS. Ubiquitination is also a well-studied PTM, and it has been reported that MAVS can be ubiquitinated. For example, the PSMA7 subunit of the 20S proteasome physically interacts with MAVS to induce ubiquitination, resulting in the induction of proteasome-mediated degradation of MAVS [38]. In our preliminary study, we observed no ubiquitination of WT MAVS or MAVS SNPs under unstimulating conditions (data not shown). Therefore, the PTMs that are involved in the MAVS SNPs that we analysed remained unclear in the present study. Further studies will determine the optimal PTM for each MAVS SNP. In contrast, the G194R SNP, located in an uncharacterized domain showed enhanced antiviral signalling. G194R is unlikely to affect the PTM of MAVS, which might explain the constitutive activation of MAVS by conformational change or aggregation [39].

Deficiency of TRAF proteins prevents activation of antiviral effects in response to dsRNA [40,41], indicating the importance of these interactions in RLR signalling. Our results were unable to show a relationship between the interaction of TRAF2 with MAVS and RLR signalling. In addition, it seemed that hyper-interaction of TRAF6 with R65W negatively regulated RLR signalling. Although R65W is located in the CARD domain of MAVS, R65W might affect RLR signalling through interaction with TRAF6. We observed that the SNPs that we tested did not alter the interaction of MAVS and RIG-I-CARD. Our data thus show that MAVS SNPs do not affect the physical interaction between MAVS and RIG-I or TRAFs. Therefore, no positive or negative association of MAVS SNPs, except R65W, with RLR signalling was found in this study. Another TRAF, such as TRAF3 or TRAF5, might instead be involved with these SNPs in MAVS.

The fact that the allele frequency distribution of SNPs differs between populations has been shown by a previous study of the rs12979860 polymorphism of the IL28B gene, which showed a striking global pattern that the C allele frequency was 100% in several East Asian populations but only less than 40% in certain African populations [42]. Allele A of rs671 in the ALDH2 gene also exhibited a certain frequency in Japanese individuals but was nearly absent in Europeans according to data published in the NCBI database (http://www.ncbi.nlm.nih.gov/projects/SNP/snp_ref.cgi?rs=671). Similarly, this difference in distribution is also observed for MAVS SNPs. For example, the C79F allele has been detected in African Americans, but not in European Americans [21]. This finding may explain why the MAVS SNPs genotyped in this study are not present in the Japanese population.

In conclusion, our study identified new functional alterations in antiviral signalling based on MAVS polymorphisms. These MAVS SNPs were not detected in a Japanese population assessed by mass examination. Although we failed to assess the risk of viral infection based on MAVS variations, we speculate that such variations may result in serious immune-related diseases.

## Supporting Information

S1 Fig Phosphorylation of MAVS. HeLa cells were transfected with an empty plasmid (mock) or a plasmid encoding WT or the indicated variant MAVS and were then incubated for 24 h. Ten micrograms of the lysates was subsequently subjected to Phos-Tag PAGE or SDS-PAGE and then blotted with anti-Myc antibody. The results are representative of three independent experiments.
(TIF)
