# Supplementary figures and images for: Alteration of Antiviral Signalling by Single Nucleotide Polymorphisms (SNPs) of Mitochondrial Antiviral Signalling Protein (MAVS)
Source: PLoS One. 2016 Mar 8;11(3):e0151173. doi: 10.1371/journal.pone.0151173 (PMC4783065; doi:10.1371/journal.pone.0151173)

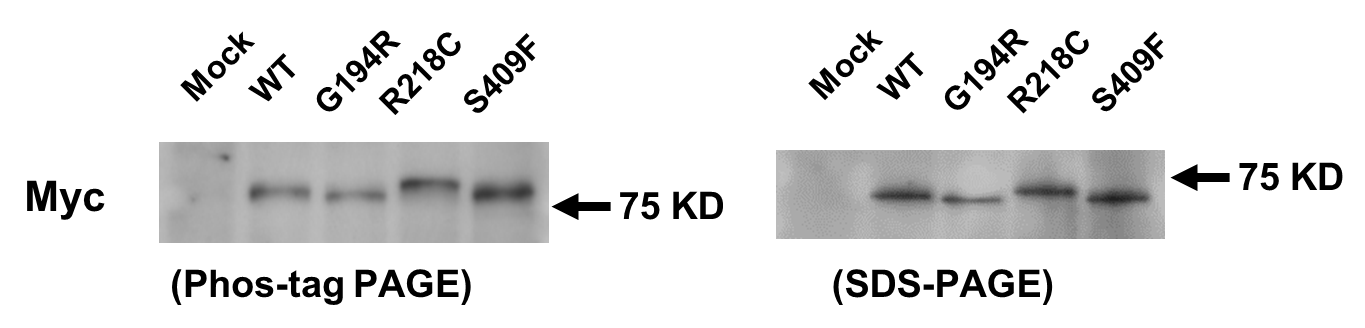

Supplement: S1 Fig — HeLa cells were transfected with an empty plasmid (mock) or a plasmid encoding WT or the indicated variant MAVS and were then incubated for 24 h. Ten micrograms of the lysates was subsequently subjected to Phos-Tag PAGE or SDS-PAGE and then blotted with anti-Myc antibody. The results are representative of three independent experiments. (TIF) [file pone.0151173.s001.tif]
